# The severity of fall injuries in Saudi Arabia: a cross-sectional study

**DOI:** 10.11604/pamj.2020.36.152.23944

**Published:** 2020-07-05

**Authors:** Mohamed Abdel Razik, Faisl Abdulmohsin Alslimah, Khalid Saeed Alghamdi, Mohammed Abdulaziz Altamimi, Adel Ahmed Alzhrani, Naif Mutrik Alqahtani, Sami Munahi Alshalawi

**Affiliations:** 1 General Surgery Department, College of Medicine, Prince Sattam Bin Abdulaziz University, Al-Kharj, Saudi Arabia,; 2 College of Medicine, Prince Sattam Bin Abdulaziz University, Al-Kharj, Saudi Arabia

**Keywords:** Accidental falls, fall injuries, injury severity score, Glasgow coma scale, hypovolemic shock, Saudi Arabia

## Abstract

**Introduction:**

fall injuries constitute a major public health concern worldwide, contributing to over 646,000 deaths every year. The aim of this study was to determine the nature and severity of fall injuries at a tertiary hospital in the Kingdom of Saudi Arabia (KSA).

**Methods:**

we conducted a cross-sectional study at the King Khalid Hospital and Prince Sultan Centre for Health Care in Al Kharj. We recruited the patients and followed them through the triage, admission and discharge processes. We analyzed the participant´s clinical notes on the electronic health record (EHR) to obtain information relevant to the study, including the nature, cause, mechanism of injury, demographic characteristics and prognostic factors captured through the injury severity score (ISS), the Glasgow coma scale (GCS) and the presence or absence of shock.

**Results:**

of 264 patients, most of the patients were children under the age of ten (25.7%), followed by young adults between the ages of twenty-one and thirty (18.2%). The ISS was associated with severe head, chest, skull, brain, scalp, rib, abdominal, pelvic and lower limb injuries. The GCS was associated with severe the head, chest, skull, brain and rib injuries (p<0.005). The degree of shock was also significantly associated with pelvic, head, chest, skull, brain, scalp, abdominal and upper limb injuries (p<0.05). **Conclusion:** fall injuries in our setting are severe. Training of staff should prioritize head, chest, skull, brain, abdominal and rib injury management. As a reference hospital, minor injuries are more likely to be managed at lower levels of care.

## Introduction

Fall injuries are one of the major public health concerns worldwide. They stand second (after road injury) among the leading causes of accidental deaths, contributing to 646,000 deaths every year [1]. Approximately 37.3 million falls require medical attention per year [2]. Elderly people are at the risk of the greatest number of falls, and about 28-35% of people above sixty-five experience falls every year [3]. Only the United States (U.S.) spent 49.5 billion dollars on the management of non-fatal and fatal falls among older adults in the year 2015 [4]. Falls are responsible for more than half of injury-related hospital admissions among older adults [5]. In Saudi Arabia, up to 49.9% of elderly people experience falls each year, resulting in fractures and traumatic brain and limb injuries [5]. Falls may cause serious injuries leading to physical and psychological disability along with a reduced quality of life. The severity of falls is directly related to the height and landing surface [6]. However, falls from low-or high-levels can cause severe injuries. In the case of low-level falls, elderly people are at a higher risk of hospitalization, longer hospital stays and increased mortality compared to that observed in younger people [6]. High-level falls are associated with severe and multiple injuries [7]. They can use a Glasgow coma scale (GCS), injury severity score (ISS) and the degree of shock to evaluate the severity of fall injuries or trauma patients [8].

Basically, GCS is one of the common scoring systems used to gauge the severity of patients with traumatic brain injuries. First published in 1974, GCS is a reliable and objective assessment of the conscious level; especially, among the patients with clinical brain injury [9]. Although GCS is a significant prognostic factor; however, it is not the gauge for brain injury only since various other factors such as cerebral perfusion and intoxication can influence it [10]. However, GCS can be used to assess brain injuries or conscious levels in patients with head injuries due to falls [11]. Injury severity score (ISS) is an anatomical scoring system that offers an overall assessment of the patients with multiple injuries. It has a linear association with morbidity, mortality, hospital stay and severity [12]. Hence, ISS can be used to assess the severity of the patients with fall injuries. Similarly, shock is an indicator of life-threatening traumatic injury as it manifests the circulatory failure. Therefore, patients with shock are immediately transported to the highest level of trauma care directly from the triage room [13]. Immediate identification of shock in trauma patients is vital to prevent multi-organ failure. Therefore, assessment of the degree of shock in traumatic fall injuries is essential in terms of management strategies. There is apaucity of literature on falls and their severity among the Saudi population. Therefore, we conducted this cross-sectional study to determine the severity of falls among the Saudi population captured through the GCS, ISS and the degree of shock irrespective of age and gender.

## Methods

This study aimed to determine the nature and severity of fall injuries at a tertiary hospital in the Kingdom of Saudi Arabia (KSA). We conducted this cross-sectional study at King Khalid Hospital and Prince Sultan Centre for Health Care (KKH&PSCHC) in Al Kharj, recruiting 264 patients. The sample size was calculated usingthe online sample size calculator open epi [14]. Taking the proportion ISS >15 in patients with fall, 18.7% of which required us to recruit at least 234 patients in order to gain a 95% confidence interval with a 5% margin of error [7]. Fall was defined as “an event which causes a person to come to rest unintentionally to ground, floor or lower level [1]. The inclusion criteria for the study were as follows: male and female genders, all ages, the provision of informed consent by the patient or the legal guardian, residing within KSA, having presented to the KKH&PSCHC and having at least one fall injury regardless of concomitant pathology. The exclusion criteria for the study were as follows: minors who failed to provide legal guardian consent and patients who had deceased in the emergency setting after the presentation and not admitted to the hospital's inpatient facility. All the patients who had deceased at the presentation or during the in-hospital stay or those who presented outside of the stipulated study period were excluded from the study. We did not consider injuries sustained prior to the fall during the study.

The proposal of the study was approved by the ethical board of the hospital. A printed informed consent form explaining inclusion in the study and assurance of confidentiality was signed by each participant before being included in the study. All the patients recruited for the study were identified at the point of presentation to the emergency department and followed through the triage, admission and discharge processes. The researchers obtained and analyzed the participant´s clinical notes on the electronic health record (EHR) to obtain information relevant to the study. This information included but was not limited to, the nature and cause of the fall injury, as well as the mechanism of injury, demographic information and other prognosticating factors. These prognosticating factors included the injury severity score (ISS), the Glasgow coma scale (GCS) and the absence or presence of degree of shock. Data were analyzed using the statistical software SPSS 22. Descriptive statistics were used to summarize means, medians, frequencies for continuous variables. The Chi-square test of independency was used to evaluate associations between categorical variables of interest. All P values less than 0.05 were considered statistically significant.

## Results

A total of 264 patients presented to the King Khalid Hospital and Prince Sultan Centre for Health Services between January 2019 and January 2020 with injuries secondary to a fall. Most of these patients were children under the age of 10 (25.7%), followed by young adults between the ages of 20-30 (18.2%) years. The vast majority (77.2%) of patients were males. We have summarized other demographic characteristics pertaining to the patients in Table 1. We categorized GCS into mild (>13) and moderate (9-12) in terms of severity. No patient wasobserved with GCS below 9 in the study. However, associations were observed between injury type and GCS. The GCS was significantly associated with head, skull and brain injuries (p<0.001). Similarly, the GCS was also have significant associationwith the chest (p<0.001) and rib (p=0.005) injuries. Similarly, GCS was significantly associated with head, chest, skull, brain and rib injuries. Specifically, we observed a lower GCS with the presence of the mentioned injuries (Table 2). Unlike the GCS, the ISS differed significantly amongst the different age groups. In terms of severity, we categorized the ISS as mild (<9), moderate (9-15) and severe (≥16). The highest median ISS amongst males age 20-30 was (16) and the highest median ISS amongst females was in the 40-50 age group with ISS of 16. The lowest median ISS amongst males was in the 30-50 age group with ISS (9). The lowest median ISS amongst females were in the twenty to thirty age group with ISS (0) and the zero to twenty age group with ISS (9) as well as those above sixty had ISS of (9). We observed associations between the ISS and the type of injury/region of injury. The severity of injuries in terms of ISS was significantly associated with the head, chest, skull, brain, scalp, rib, abdomen, pelvic and lower limb injuries (p< 0.05). Specifically, a higher ISS was associated with the presence of the mentioned injuries. These are delineated in Table. The mean GCS score did not vary significantly between the different age groups. Indeed, the mean GCS score fluctuated between 14 and 15 for all age brackets, regardless of gender. The degree of shock significantly associated with pelvic, head, chest, skull, brain, scalp, abdominal, upper limb and other injuries (p<0.05). ISS scores were also higher in patients with these injury types (Table 3). Similarly, we analysed the relationship between the injury type and shock. These findings are summarized in Table 4.

**Table 1:** demographic characteristics of the study population

Demographic Characteristics		Frequency	Percentage
Age (In Years)	0 To 10	52	19.7
	10 To 20	40	15.2
	20 To 30	44	16.7
	30 To 40	48	18.2
	40 To 50	36	13.6
	50 To 60	32	12.1
	Above 60	12	4.5
Gender	Female	60	22.7
	Male	204	77.3
Causes	Fall from height	256	97.0
	Fall from height on sharp object	8	3.0
Degree of shock	1^st^ degree	20	7.6
	2^nd^ degree	4	1.5
	3^rd^ degree	240	90.9
GCS Score	Mild	256	96.97
	Moderate	8	3.03
ISS score	Mild	152	57.58
	Moderate	68	25.76
	Severe	44	16.67
Total		264	100.0

**Table 2:** associations between ISS and injury type/region

Type of injuries		ISS Score			Total	P values
		Mild	Moderate	Severe		
Head injury	No	140( 66.04)	48 (22.64)	24 (11.32)	212(100.0)	<0.001*
	Yes	12 (23.08)	20 (38.46)	20 (38.46)	52(100.0)	
Chest injury	No	132(57.89)	68 (29.82)	28 (12.28)	228(100.0)	<0.001*
	Yes	20 (55.56)	0 (0.00)	16 (44.44)	36(100.00)	
Skull fracture	No	140(61.40)	52 (22.81)	36 (15.79)	228(100.0)	0.004*
	Yes	12(33.33)	16(44.44)	8(22.22)	36(100.0)	
Brain damage	No	152 (62.30)	60(24.59)	32(13.11)	244(100.0)	<0.001*
	Yes	0(0.00)	8(40.00)	12(60.00)	20(100.00)	
Scalp laceration	No	152(59.38)	64(25.00)	40(15.63)	256(100.0)	0.001*
	Yes	0(0.00)	4(50.00)	4(50.00)	8(100.00)	
Rib fracture	No	132( 55.93)	68(28.81)	36(15.25)	236(100.0)	<0.001*
	Yes	20(71.43)	0(0.00)	8(28.57)	28(100.00)	
Abdomen laceration	No	152(60.32)	64(25.40)	36(14.29)	252(100.0)	<0.001*
	Yes	0(0.00)	4(33.33)	8(66.67)	12(100.0)	
pelvis injury	No	148(62.71)	56(23.73)	32(13.56)	236(100.0)	<0.001*
	Yes	4(14.29)	12(42.86)	12(42.86)	28(100.00)	
pelvis fracture	No	148(61.67)	56(23.33)	36(15.00)	240(100.0)	<0.001*
	Yes	4(16.67)	12(50.00)	8(33.33)	24(10.00)	
pelvis laceration	No	152(58.46)	68(26.15)	40(15.38)	260(100.0)	0.001*
	Yes	0(0.00)	0(0.00)	4(100.00)	4(100.00)	
Lower limb	No	104(50.98)	68(33.33)	32(15.69)	204(100.0)	<0.001*
	Yes	48(80.00)	0(0.00)	12(20.00)	60(100.0)	
Total		152(57.58)	68(25.76)	44(16.67)	264(100.0)	
Frequency (%), *: Significant, ISS: Injury severity scale						

**Table 3:** associations between GCS and injury type

Type of injuries		GSC Score			P values
	****	**Mild**	**Moderate**	**Total**	****
Head injury	No	212(100.00)	0(0.00)	212(100.0)	<0.001*
	Yes	44(84.62)	8(15.38)	52(52.00)	
Chest injury	No	228(100.00)	0(0.00)	228(100.00)	<0.001*
	Yes	28(77.78)	8(22.22)	36(100.00)	
Skull fracture	No	228(100.00)	0(0.00)	228(100.0)	<0.001*
	Yes	28(77.78)	8(22.22)	36(100.0)	
Brain damage	No	240(98.36)	4(1.64)	244(100.0)	0.001*
	Yes	16(80.00)	4(20.00)	20(100.0)	
Rib fracture	No	232(98.31)	4(1.69)	236(100.0)	0.005*
	Yes	24(85.71)	4(14.29)	28(100.0)	
Total		256(96.97)	8(3.03)	264(100.0)	
Frequency (%), *: Significant, GCS: Glasgow Coma Scale					

**Table 4:** associations between the degree of shock and type of injury

Shock degree	Pelvis fracture			Pelvis laceration		
	**0**	**1**	**Total**	**0**	**1**	**Total**
1^st^ degree	12(5%)	8(34%)	20	16(6%)	4(100%)	20
2^nd^ degree	4(1%)	0(0%)	4	4(1%)	0(0%)	4
3^rd^ degree	224(94%)	16(66%)	240	240(92%)	0(0%)	240
Total	240 (100%)	24 (100%)	264	260 (100%)	4 (100%)	264
****	P value<0.001*	****	****	P value<0.001*	****	****
****	**Head injury**	****	****	**Chest injury**	****	****
****	**0**	**1**	**Total**	**0**	**1**	**Total**
1^st^ degree	12 (6%)	8 (15.3%)	20	12(5%)	8(15%)	20
2^nd^ degree	0 (0.0%)	4 (7.7%)	4	0 (0.0%)	4 (7%)	4
3^rd^ degree	200 (94%)	40 (76.9%)	240	216 (94%)	24(77%)	240
Total	212(100%)	52(100%)	264	228(100%)	36(100%)	264
****	P value<0.001*	****	****	P value<0.001*	****	****
****	**Skull fracture**	****	****	**Brain damage**	****	****
****	**0**	**1**	**Total**	**0**	**1**	**Total**
1^st^ degree	20 (8%)	0(0%)	20	16	4(20%)	20
2^nd^ degree	0 (0%)	4(11%)	4	0 (0%)	4 (20%)	4
3^rd^ degree	208(91%)	32 (88%)	240	228 (93%)	12 (60%)	240
Total	228(100%)	36(100%)	264	240(100%)	20(100%)	264
****	P value<0.001*	****	****	P value<0.001*	****	****
****	**Scalp laceration**	****	****	**Abdomen laceration**	****	****
****	**0**	**1**	**Total**	**0**	**1**	**Total**
1^st^ degree	16 (6%)	4(50%)	20	8(3%)	12(100%)	20
2^nd^ degree	4 (1%)	0 (0%)	4	4 (1%)	0(0%)	4
3^rd^ degree	236(92%)	4(50%)	240	240(95%)	0(0%)	240
Total	256100%)	8(100%)	264	252(100%)	12(100%)	264
****	P value =0.003*	****	****	P value<0.001*	****	****
****	**Pelvis injury**	****	****	**Other injury**	****	****
	0	1	Total	0	1	Total
1^st^ degree	8(3%)	12(42%)	20	12(5%)	8(40%)	20
2^nd^ degree	4 (1%)	0 (0%)	4	4(2%)	0(0%)	4
3^rd^ degree	224(95%)	16(58%)	240	228(94%)	12(60%)	240
Total	236(100%)	28(100%)	264	244(100%)	20(100%)	264
****	P value<0.001*	****	****	P value<0.001*	****	****
****	**Upper limb injury**	****	****	****	****	****
****	**0**	**1**	**Total**	****	****	****
1^st^ degree	20(12%)	0(0%)	20			
2^nd^ degree	0(0%)	4(4%)	4			
3^rd^ degree	144(87%)	96(96%)	240			
Total	160(100%)	100(100%)	264			
	P value<0.001*					


P value<0.05* =statistically significant association between injury and shock degree; P value>0.05 =no association between injury and shock degree

## Discussion

Fall injuries in our study setting are severe captured through the GCS, ISS and the degree of shock. The injury severity score (ISS) was significantly higher in those with head, chest, skull, brain, scalp, spine, rib, abdomen, pelvic and lower limb injuries. Glasgow coma scale (GCS) was significantly lower in those with head, chest, skull, brain and rib injuries. Similarly, the degree of shock was more pronounced in those with pelvic, head, chest, skull, brain, scalp, abdominal, upper limb and other injuries. Turgut *et al*. [15] conducted a retrospective study at Inonu University in Turkey including 460 patients with fall from height and reported that ISS had significant effect on mortality. Another study has reported a linear association of ISS with morbidity, mortality, hospital stay and severity [12]. These studies demonstrate that ISS can be used to assess severity of fall injuries. Aunon-Martin *et al*.[16] reviewed the severity of 188 patients who sustained a high fall in terms of ISS and new injury severity score (NISS). They reported a significant association between the height of the fall, ISS and NISS since the height is associated with severe blunt injuries. Recently, Huang *et al*.[17] conducted a retrospective study including 938 patients in order to determine the capacity of GCS, ISS and revised trauma score (RTS) in young children with trauma. They reported increased mortality, longer hospital and prolonged intensive care unit (ICU) stay with worse ISS, GCS and RTS. In addition, they demonstrated ISS as the best predictor of mortality among patients with trauma. In this context, the present study reveals that serious fall injuries were associated with worse ISS. Similar to the results of the present study, Wong *et al*. [15] reported a significant effect of scoring systems, injuries of head, skull, spine and thorax on mortality.

Clinical severity and outcome of traumatic brain injuries are frequently defined by GCS. However, the use of GCS ≤12 in traumatic brain injury is of limited value in patients with multiple injuries [18]. Moreover, the components of GCS have a different effect on the sum of GCS and consciousness [19]. To avoid this discrimination, the combination of different scoring systems may offer greater reliability regarding the assessment of the severity of the injuries. Therefore, the present study used the GCS, ISS and the degree of shock to determine the severity, and these indicators supported each other in most of the injuries. Age is an important factor in clinical research. Kehoe *et al*. [20] used a national trauma database to identify the effect of age on GCS of the patients with the equivalent severity of the intracranial injury. They reported that GCS was higher in older patients than in the younger ones. However, the present study revealed no significant effect of age on GCS. Similarly, GCS has prognostic value in patients with chest injuries since GCS ≤8 is an independent risk factor for prolonged mechanical ventilation [21]. Hypovolemic, hemorrhagic, or circulatory shock is critical in any traumatic injury requiring immediate resuscitation. Pelvic fractures with hemodynamic instability are associated with increased mortality [22]. Traumatic hemorrhagic shock leads to 30-40% deaths within 24 hours of the onset of injury [23]. Therefore, immediate identification and prompt resuscitation are essential in such patients. To the best of our knowledge, the present study is the pioneer study to evaluate the severity of fall injuries among the Saudi population. The strength of the study is that it has reported the scoring systems (ISS, GCS and degree of shock) concerning the location of fall injuries - the forgotten area of clinical research. The limitation of the study is that it has not included outcome and mortality for fall injuries, which warrant further research in the future.

## Conclusion

Falls may resulting in severe head, chest, spine, abdominal, pelvic and limb injuries were the most frequent in our setting. Severe falls can be assessed using the ISS, GCS and the degree of shock. These scoring systems complement each other, especially in patients with multiple injuries and are beneficial in appropriate patient assessment and monitoring.

### What is known about this topic

- Fall injuries are one of the major public health concerns worldwide, contributing to over 646,000 deaths every year;
- Approximately 37.3 million falls require medical attention per year;
- In Saudi Arabia, up to 49.9% of elderly people experience falls each year, resulting in fractures and traumatic brain and limb injuries.

### What this study adds

- Falls result in severe injuries of head, chest, spine, abdomen, pelvis and extremities in our setting;
- Our findings support the use the Glasgow coma scale, the injury severity score and the degree of shock to gauge the severity of fall injuries;
- The injury severity score is more stable in capturing injury severity compared to the Glasgow coma scale.
